# Histological Processing of Scaffolds: Challenges and Solutions

**DOI:** 10.3390/jfb16080279

**Published:** 2025-07-31

**Authors:** Tomas Ragauskas, Ilona Uzieliene, Eiva Bernotiene

**Affiliations:** 1 Department of Regenerative Medicine, State Research Institute Centre for Innovative Medicine, Santariskiu Str. 5, 08406 Vilnius, Lithuania; ilona.uzieliene@imcentras.lt (I.U.); eiva.bernotiene@imcentras.lt (E.B.); 2 Faculty of Fundamental Sciences, VilniusTech, Sauletekio Av. 11, 10223 Vilnius, Lithuania

**Keywords:** scaffolds, histology, FFPE, cryomicrotomy, vibrating microtomy

## Abstract

Scaffolds are widely used in bioengineering, both as 3D native tissue-mimicking models for investigating mechanisms under physiological and pathological conditions and also as implantable agents in regenerative medicine. Histological approaches, mainly formalin-fixed paraffin-embedded (FFPE) and frozen sample sectioning, are commonly applied to evaluate cell distribution and tissue-like properties of scaffolds. However, standard histological processing is not always compatible with the materials that scaffolds are made of. Thus, some adaptations to protocols are required to obtain intact sections. In this review we discuss challenges related to the histological processing of scaffolds and solutions to overcome them. We sequentially cover processing steps of the three main histological techniques for sample preparation—cryomicrotomy, FFPE samples microtomy and vibrating microtomy. Furthermore, we highlight the critical considerations in choosing the most appropriate method based on scaffold composition, mechanical properties and the specific research question. The goal of this review is to provide practical guidance on choosing reliable histological evaluation of complex scaffold-based systems in tissue engineering research.

## 1. Introduction

Scaffolds hold promise in regenerative medicine, acting as a supportive system to improve therapeutic potential and targeted delivery of cells, growth factors, nanoparticles and other bioactive agents [1,2,3]. Their numerous distinguished properties are directed towards clinically relevant implants in various tissues, cartilage being arguably one of the main targets due to the big potential in scaffold-based rheumatic disease treatment applications. Given the pathological nature of these ailments affecting the whole joint, the need to recapitulate the so-called osteochondral unit is directing researchers to the creation of multiphasic designs, mimicking distinct cartilage layers as well as subchondral bone. A number of techniques are used to produce non-homogeneous constructs with gradient porosity, 3D printing and multilayering approaches representing the most advanced methods to date [4].

**Figure 1:**
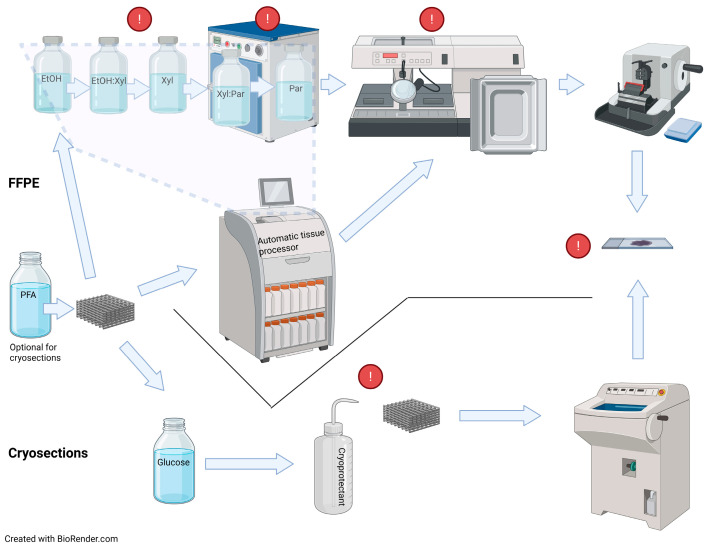
Schematic representation summarizing processing steps for standard formalin-fixed paraffin-embedded (FFPE) and frozen samples sectioning, highlighting steps where some scaffold-specific modifications usually should be applied (exclamation marks). EtOH, ethanol; Par, paraffin; PFA, paraformaldehyde; Xyl, xylene.

Histological evaluation of implantable biodegradable scaffold constructs is commonly performed using standard formalin-fixed paraffin-embedded (FFPE) and frozen samples histological protocols [5,6,7,8,9]. However, often integration of layers in multiphasic scaffolds and/or cell distribution and extracellular matrix formation within should be evaluated beforehand. Ultrastructure of pure scaffolds (without biological constituents) is observed by means of scanning electron microscopy or other techniques used in material sciences. Biocompatible scaffold models are by nature the result of biomaterial engineering, and while standard histological procedures may be applicable to some materials, in most cases, at least some adaptations are required at one or more processing steps (Figure 1). Although numerous publications review the fabrication and modification of synthetic scaffolds, their functional properties and application in biomedicine [10,11,12], there is, to the best of our knowledge, a lack of comprehensive overviews analyzing the histological challenges in scaffold-based tissue engineering, except from a few studies focused on designing scaffold-specific protocols [13,14,15,16]. In this review, we discuss challenges related to scaffolds, including hydrogels, histological processing and means to overcome them. The three most widely used histological techniques are covered in a sequential manner of the processing steps.

## 2. Sample Fixation for Further Processing

Fixation is the first and most critical step in histology. The structure of samples prepared for cryosectioning, vibrating microtomy and especially FFPE sample sectioning is routinely preserved by fixatives, the most widely used being paraformaldehyde (PFA), which cross-links proteins and protects tissues throughout subsequent processing steps [17]. Other fixatives, based on distinct mechanisms of action—ketones, acids, alcohols and HOPE (Hepes-glutamic acid buffer-mediated Organic solvent Protection Effect) [18,19]—are commonly used as well. Counterintuitively, fixatives could also disrupt internal structures of scaffolds. It was shown that 4% PFA had a more pronounced effect on collagen hydrogel compared with 2% PFA [20]. On the other hand, paraformaldehyde and glutaraldehyde are among the chemicals used to cross-link gelatin fibers, a critical step in producing gelatin hydrogels (uncross-linked gelatin dissolves at low temperatures) [21], and thus should also be compatible with gelatin methacryloyl [22,23], widely used in photoinducible scaffold polymerization.

The fixation of hydrogel-based scaffolds possesses additional challenges, especially those that are ionically cross-linked. As assessed by Da Silva et al. (2023), after 24 h of incubation, traditional aldehyde-based fixatives (10% neutral-buffered formalin (NBF), 4% PFA and glutaraldehyde) dissolved or distorted calcium alginate hydrogels, probably due to the formation of calcium and phosphate (from the buffer) precipitate and subsequent decrease in cross-linking [24]. Prolonged cross-linking with calcium chloride or the addition of it to the fixative buffer improved preservation of the hydrogel structure. Authors also tested formalin-free alcohol-based (>30% ethanol) commercial fixative, which they concluded yielded the best results [24]. Consistently with the above-mentioned study, the structure of alginate/gelatin beads was preserved by their preincubation in barium chloride before incubation in NBF [25]. Barium ion is also a divalent cation and a strong alginate cross-linker [26].

Notably, the fixative should be compatible with the stains used later. For example, methanol-containing fixatives (such as NBF) could interfere with actin staining [27]. Also, after prolonged fixation, as is the case with paraffin-embedded samples, the fixative should be carefully washed out.

## 3. Cryomicrotomy

### 3.1. Cryopreservation

**Figure 2:**
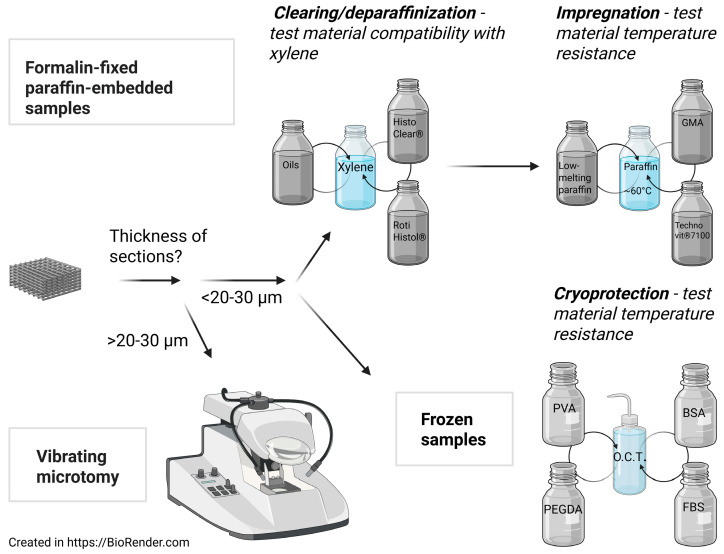
Workflow of scaffold histological processing highlighting proposed considerations for material testing and some of the alternatives for common reagents (shown in gray). BSA, bovine serum albumin; FBS, fetal bovine serum; GMA, glycol methacrylate; O.C.T., optimal cutting temperature; PEGDA, polyethylene glycol diacrylate; PVA, polyvinyl alcohol.

Cryosectioning requires minimal specimen processing compared with FFPE sample preparation; thus, numerous studies employed this technique for various scaffolds, such as poly (lactide-co-glycolide) (PLGA) [28] and polycaprolactone (PCL) [29,30,31]. Although cryosectioning is a relatively straightforward method to achieve sections, proper cryopreservation is of high importance for both biological tissues and synthetic scaffolds, especially given that in many protocols, proper freezing acts as a fixative itself [32,33] (Figure 1). Unless rapid freezing techniques are applied, ice crystals readily form at low freezing rates, usually used to embed samples in cryopreservation media [34]. Fine fibers of scaffolds are highly prone to such damaging effects. Although sucrose, as well as other cell-permeating (propanediol, ethylene glycol, dimethyl sulfoxide (DMSO)) and non-permeating (trehalose, glucose, polyvinylpyrrolidone, polyethylene glycol (PEG)) agents, prevent the formation of nucleation sites required for ice crystal formation [35] and are frequently used as a first-choice cryoprotectant, it is not always perfectly suited for scaffolds containing water-rich structures, such as hydrogels. Ruan et al. (2013) compared the cryoprotective potential of 14 protein-based and nonprotein-based solutions, which were tested on PEG hydrogel [36]. Some infiltration solutions (30% sucrose and 30% glycerol) resulted in a shattered hydrogel; others (100% DMSO and 100% PEG) caused separation of the hydrogel from the surrounding block due to shrinkage and changes in elasticity [36]. Still other solutions (100% normal goat serum, nonfat dry milk, 10% bovine serum albumin (BSA), 10% and 30% Ficoll) affected the integrity of the hydrogel construct, possibly due to incomplete penetration. And only five infiltration solutions (100% fetal bovine serum, 30% BSA, 1% polyvinyl alcohol (PVA), optimal cutting temperature (O.C.T.) and Fisher cryogel) produced complete sections [36] (Figure 2). Some studies also reported using cryopreservation media for cryosectioning, such as O.C.T. for PCL/poly (ethylene)oxide (PEO; used to increase porosity) [37] and tissue freezing media (Fisher) for PLGA [14] scaffolds, even though additional steps for better infiltration are not stated. Infiltration efficacy may depend on the porosity of the sample; thus, no additional steps may be required in some cases. It should be noted that both O.C.T. and Fisher freezing media contain PVA (~10%), which seems to confer the main protective effect. Also, other components may provide additional protection against brittleness, which is a common problem in cryosectioning. Moreover, the quality of media infiltration is of critical importance; thus, prolonged infiltration time or its enhancement using vacuum assistance [14] is often applied. Although widely used, cryoprotective media may not be a universal solution to all samples, especially thinner ones, as was demonstrated by the study of Cocco et al. (2003), who tested several concentrations of PVA on tissues and allowed sectioning up to 1.5 µm thickness (Figure 2) [38]. The authors propose that the optimal concentration of PVA helps match the refreezing rate of the medium and specimen caused by a short melting phase after hitting the edge of the blade. The study also underlines the importance of cutting temperature on section integrity. Maintaining a low temperature of blades (≤−33 °C) compensates for the relatively high temperature of the specimen (−1 °C–−4 °C), required to prevent cracks (especially, between tissue and media) in thicker specimens (20–100 µm). The authors also found that, although PEG alone did not work as cryoprotectant, it proved to be an effective plasticizer preventing fractures and cracks in the tissue [38]. An alternative to embedding in PVA-containing media is spraying PVA before each stroke of cryomicrotome to cover the specimen with a thin protective layer, which allows for the cutting of thinner sections (of several micrometers), avoiding curling and maintaining integrity [39].

Hydrogels, which are widely used to improve the elasticity of scaffolds, present an additional challenge due to their high water content. Sugar-based cryopreservation usually results in brittle embedding blocks and difficulties in cryosectioning [40], although sucrose was used to protect hyaluronic acid (HA) hydrogel, followed by snap-freezing [41]. To preserve the microstructure of intestinal villus-like polyethylene glycol diacrylate (PEGDA)-based scaffolds, Altay et al. (2020) embedded them in a photopolymerizable lower-molecular-weight PEGDA hydrogel, which provided hard encapsulation [42]. Subsequent re-embedding into O.C.T. (for cryosectioning) or agarose (for vibrating microtomy) both resulted in good-quality sections [42].

As synthetic scaffolds and hydrogels are usually characterized by their small-sized pores, cryoprotectant infiltration effectiveness is of high importance. Commonly used O.C.T. is quite viscous for rapid sample penetration. To this end, Yang et al. (2007) further improved O.C.T. infiltration to collagen hydrogel by first applying several rounds of 20% sucrose infiltrations and, on the subsequent day, infiltration with an increasing ratio of O.C.T. diluted in sucrose [43]. Similar protocol was applied to PEGDMA hydrogels [44], HA scaffolds [45] and implantable polylactic acid (PLA)-based osteochondral constructs [46].

### 3.2. Cryosectioning

Followed by cryopreservation and prior to sectioning, samples should be embedded in proper medium, commonly O.C.T., which gives samples a supportive structure during sectioning. However, standard microscopy slides, well suited for biological specimens, often fail with synthetic materials. Poor adhesion of scaffold-containing sections is an issue of concern (Figure 1). Brown et al. (2005) compared O.C.T. and gelatin as embedding media for polyglycolic acid (PGA) and PLGA scaffolds and found that for thicker sections (10 and 25 µm), both media work well, while only O.C.T. constantly preserved intact thin sections (5 µm) [47]. However, almost all 10 µm thickness O.C.T.-embedded sections detached from slides after 2 min rinsing, in contrast to gelatin-embedded, virtually all of which remained attached [47]. Gelatin has a slightly negative charge, which may aid adhesion to the positively charged surface of slides. Keeping acid-cleaned slides with sections at 50 °C overnight proved to help maintain PLGA scaffolds attached [14]. This same study also provided another hint by utilizing histological staining in situ (before sectioning), thus avoiding extensive rinsing and incubation in solutions when there is the biggest risk of section loss.

## 4. Vibrating Microtomy

Vibrating microtomy is mostly used to section fragile tissues with minimal processing and could provide several important advantages over other methods. First of all, it enables thicker sectioning, usually from 30 µm up to one millimeter, while it proves difficult to obtain paraffin or cryosections thicker than 20 µm [48] (Figure 2). Thicker sections may be advantageous, for example, when assessing cell distribution within the scaffold. Secondly, besides agarose-embedding, this technique does not require any additional processing of the sample, and using low-melting agarose also prevents temperature-related issues. Given that agarose does not infiltrate the sample and forms the supportive surrounding, it helps stabilize the structure of commonly fragile and/or elastic scaffolds, especially detachment-prone multilayered ones. Usually 2–5% agarose is enough to obtain sections that retain stability even in the case of their transfer [48]. Vibrating microtomy was successfully used for sectioning of collagen hydrogel, as this technique does not need dehydration or freezing steps, both of which could disrupt the structure of the scaffold [20]; however, in the case of PEGDA-based scaffold, with additional embedding [42]. Shortcomings of the technique include that distinct materials may have different compatibility with agarose. Also, this type of sectioning is limited to thicker sections, usually over 30 µm.

## 5. Formalin-Fixed Paraffin-Embedded Histology

Standard paraffin embedding, with no modifications reported, was applied for some scaffold constructs, such as silk fibroin (SF) [49], SF-hydroxyapatite [50], PCL [51] and poly (2-hydroxyethyl methacrylate) [52]. For others, only minor adaptations were applied, as for commercially available polystyrene scaffold Alvetex^®^ (Reinnervate Limited, Sedgefield, UK), where only HistoClear^®^ (National Diagnostics, Atlanta, GA, USA) was used instead of xylene [53]. However, some steps of complex FFPE samples processing are not compatible with some of the scaffold materials (Figure 1).

### 5.1. Dehydration

Dehydration in FFPE histology is a critical step required for full paraffin penetration into the tissue, as it is insoluble in water. Although biological tissues retain their structure due to the subsequent rehydration step, for some constituents of the scaffolds, dehydration with the widely used effective dehydrating agent ethanol may pose additional challenges. We observed that polypyrrole (PPy)-coated gelatin–glucose scaffolds form dense layers at the borders of a scaffold, and this effect is also seen in frozen sections when no other reagents except ethanol (for sterilization purposes) were applied. Similar black coatings of PPy were observed in optical and scanning electron microscopy images of diazonium salt of sulfanilic acid-modified but not plain silk films [54]. This may be due to the coacervation process, which induces liquid–liquid phase separation of charged molecules (as oxidized PPy is) by ethanol or salts [55,56]. Thus, in particular cases, alternatives are required. Acetone is another dehydration agent and was used to prepare poly-L-lactic acid (PLLA) scaffolds for glycol methacrylate (GMA) embedding [57].

### 5.2. Clearing

The clearing step is used to remove ethanol from the tissue and to prepare it for paraffin infiltration. As both ethanol and paraffin are soluble in xylene, it is well suited as an intermediate agent to fulfill both of the tasks. Thus, in standard protocols, sequential incubations in ethanol–xylene, pure xylene, xylene–paraffin and pure paraffin are applied. Xylene used in histological settings is usually a mixture of xylene isomers (o-xylene, p-xylene and m-xylene), and some of them, as well as the mixture itself, melt PCL [58] and also affect the structure of gelatin-based scaffolds (our observations). Therefore, other approaches are required to process xylene-soluble materials. One such mean is to avoid clearing agents completely. Automatic tissue processors, which perform dehydration, clearing and paraffin impregnation steps, employ a vacuum to infiltrate solutions into the tissue and avoid the use of xylene [59] (Figure 1). HOPE also provides an interesting clearing-free approach, in which samples, after fixation in HOPE solutions and dehydration in acetone, are directly transferred to molten paraffin [18,19]. Another mean is to use alternatives to xylene. D-limonene, a terpenoid found in citrus peels, is gaining pace as an effective clearing agent. D-limonene-based (~96%) RotiHistol^®^ (Carl Roth, Karlsruhe, Germany) and HistoClear^®^ proved to be appropriate alternatives to xylene due to much lower toxicity and comparable efficacy [29,60,61,62] (Figure 2). Dębski et al. (2022) compared the standard histological protocol with a modified one, in which the authors replaced xylene with HistoClear^®^ and also standard (57 °C melting temperature) with low-melting (49 °C melting temperature) paraffin and evaluated them on PCL scaffolds in a rat model [16]. Xylene replacement prevented PCL fiber melting and swelling, consistent with PCL insolubility in limonene [58], while low-melting paraffin also prevented swelling and expansion [16]. Our practice with RotiHistol^®^ also provided adequate clearing results of gelatin–glucose–PPy scaffolds, although it affected the structure of the scaffold (inner gelatin layer shrank and separated from border layers). Citrus essential oils, when incorporated into gelatin, are known to lower thermal degradation resistance of gelatin [63], which could affect scaffolds during temperature-sensitive steps. This reagent was used in place of xylene in regular protocols in our laboratory; only the step of mixture with 96% ethanol was skipped, as RotiHistol^®^ and ethanol do not intermix (not published).

Given the toxic nature of xylene [64], many histology laboratories are motivated to seek more human-friendly clearing agents, which could also be more compatible with scaffold histology [24]. Particular attention was paid to various biological oils (Figure 2), and numerous publications describe their effectiveness in terms of gross tissue features (translucency, shrinkage, rigidity) and staining quality, usually comparing them to xylene. Several studies exploited cedar wood oil and obtained comparable results of nuclear and cytoplasmic staining [65,66], as well as background staining and artifacts [66] to xylene. Cedar wood is viscous, thus alone is not able to infiltrate tissues, and thus should be diluted. Thamilselvan et al. (2021) applied 95% cedar wood oil diluted in xylene to several tissues, while Indu et al. (2014) report using 8% of this oil, though they have not stated the diluent used [65,66]. Bleached (heated at 50–60 °C) palm oil is also reported to achieve adequate histological and immunohistological staining quality by some studies [67], although lower than that with xylene [68] or causing poor paraffin infiltration [69] by others. Coconut oil was also tested against xylene and observed to provide similar results in terms of gross features and staining quality, and also caused less shrinkage [70]. Less shrinkage using carrot, pine and rose oils was also reported in a study by Swamy et al. (2015), possibly relating to the observed changes in rigidity of the tissues, and, furthermore, cellular morphology and staining quality were evaluated as comparable to that of xylene [71]. Andre et al. (1994) tested several oils of different saturation levels (pure clearing step was skipped, and tissues were incubated in oil–paraffin mixtures immediately after dehydration) and concluded that the quality of liver and brain sections was comparable to xylene, although with some tissue-specific differences [72]. It should be mentioned that a temperature of at least 37–40 °C (in some cases up to 60 °C) should be maintained during procedures with oils, given their higher melting point compared with xylene. N-Heptane [73], propylene glycol methyl ester (PGME) [74], butyldecanoate [69], isopropanol–mineral oil mixtures [75] and pure isopropanol [76,77] were all tested, and their clearing efficacy was concluded as suitable for the tissues evaluated. However, it should be noted that all of these studies were performed on biological tissues, and their translation to cell-seeded synthetic scaffolds should be additionally tested. Studies on the physicochemical properties of composite materials of scaffolds, such as one by Bordes et al. (2010), in which the authors tested PCL solubility in an array of chemicals, could provide valuable guidelines for their use [58]. For example, PCL was found to be insoluble in n-heptane but soluble or partially soluble in xylenes in this work [58]. On the other hand, scaffold polymers are often modified with chemical groups or other composite materials to increase their biocompatibility or other properties (elasticity, etc.) [78], prompting testing of particular composites.

### 5.3. Impregnation and Embedding

Standard paraffin infiltration and embedding protocols were used in some studies [79]; however, physicochemical properties of scaffold constituents (temperature resistance, elasticity, etc.) and pore size could determine whether the standard protocol is enough or some modifications are required, and the details for paraffin infiltration and embedding are not always specified. For instance, paraffin embedding was applied to mesenchymal stem cell-seeded PGA scaffolds without modifications [79]. The issue of infiltration was further highlighted by the study of James et al. (2004), where the authors showed reduced paraffin impregnation when they applied modified protocol (shortened times of dehydration, clearing and paraffin impregnation steps), although it preserved the structure of sodium alginate/gelatin hydrogels better, and vice versa standard protocol showed better infiltration at the cost of section integrity [13]. The best results in this study were achieved by vacuum-assisted embedding to GMA [13].

Concerning the temperature resistance issue, probably the most straightforward way is to replace standard paraffin with low-melting paraffin, which has different melting points depending on additives (usually in the range of 43–53 °C) [15] (Figure 2). In some cases, temperatures on the lower edge of the range should be obtained, especially when scaffold materials are modified to increase their biocompatibility. For instance, we observed that plasma-treated PCL scaffolds (to increase hydrophilicity) dissolved fully under much lower (below 55 °C) temperatures compared with unmodified ones. Consistently, using an automatized tissue processor and standard paraffin resulted in dissolved PCL/PLA scaffolds [15]. At least some hydrogels also suffer from temperature regime during wax impregnation (as well as from dehydration) [40]. It should be noted that the temperature during embedding should exceed that of paraffin melting at least by a few degrees; otherwise, a crust immediately forms on the top.

Some studies replaced paraffin with other alternative embedding materials, with mixed results. Treatment with 10% and 25% gelatin before embedding in standard paraffin was shown to improve the thermal stability of PCL/PLA scaffolds; however, this treatment resulted in partly melted and shrunken fibers and no utilizable sections [15]. Acrylic resin prevented PCL/PLA scaffolds from thermal degradation but could not provide sections thicker than 3 µm [15]. GMA is one of the first choices and was used for PLC [80], PLA [81,82] and PLLA [57] scaffolds (Figure 2). It was tested as infiltration and embedding media to PLA sponges (3 mm thickness). Samples were kept in aqueous GMA solutions of increasing concentrations before infiltration of GMA with the aid of catalyst benzoyl peroxide and embedding in the mixture of GMA solutions. This technique preserved the structure of the samples better compared with paraffin-embedding (xylene substitute was used), which disrupted the structure of the sponge. However, paraffin infiltration and embedding temperature (60 °C) was close to PLA glass transition temperature (62 °C), potentially having a negative effect on PLA structure, while much lower temperatures were required for GMA infiltration (4 °C) and embedding (room temperature) [81]. Another study, however, showed that GMA embedding disrupts the edges of the PLA scaffold, while methylmethacrylate resin and polyester (Castoglas) resin caused substantial folding of sections. The authors hypothesized this could be due to temperature issues, given that polymerization of these polymeric resins is exothermic. On the other hand, epoxy resin and paraffin (55 °C melting temperature) embedding displayed a clear border between them and the sample and preserved the structure of the layer [82]. The use of 2-hydroxyethyl methacrylate-based Technovit^®^ 7100 (Kulzer, Hanau, Germany) on PCL scaffolds was also reported [83] (Figure 2).

During embedding, proper orientation of the scaffold ensures the retrieval of the data needed [45]. Often, cross-sections are acquired to obtain information about layer composition and also cell distribution across the scaffold. Thus, placing thin scaffolds (usually from several hundred micrometers up to 1 mm) vertically may possess some difficulties, even given that the viscosity of both paraffin and cryogels (in the case of cryosectioning) eases the handling. Tools such as fine forceps or wooden sticks are employed; however, they could leave some traces inside rapidly solidifying paraffin and inferior sectioning later. HistoGel™ (Richard-Allan Scientific, Kalamazoo, USA) is used to embed tiny specimens to improve handling, as it can be later processed as regular biological material and also provides some structural support during sectioning [84].

### 5.4. Sectioning

Sectioning of paraffin blocks poses some challenges related to the quality of sections (rippling, disintegration, etc.) and their adhesion to the slides (Figure 1).

As is the case with cryosectioning, plasticizers are also sometimes used during embedding to improve sectioning quality. Burg et al. (1996) achieved a soft structure of embedding media methylmethacrylate by adding n-butyl phthalate [82]. Sectioning direction also could affect the quality of sections, as exemplified by acquiring flatter epoxy resin-embedded PLA scaffold sections when cut laterally compared with when cut longitudinally [82].

To improve adhesion, specially prepared (either commercially available or custom-made) histological slides with a charge-applied surface are used. For example, polylysine-coated slides improve the handling of sections of hard-to-attach tissues [85], such as cartilage [86]. Synthetic scaffolds differ from biological tissues by their physicochemical properties substantially, therefore making them even more difficult to retain, which we observed working with PCL scaffolds. Incubation of slides at a higher temperature (about 40 °C) [19] removes water leftovers and slightly melts paraffin, which aids in adhesion. Moreover, GMA assists in section adhesion to the slides, due to its hydrophilicity [87].

### 5.5. Deparaffinization

Xylene is commonly used to remove the paraffin but also makes sections more transparent, which is critical for later visualization. In fact, other substances could perform this step, primarily oil-based, at least partly due to some oils having a similar refractive index to lipids [88]. An increasing number of studies report using xylene substitutes [89]. HistoClear^®^ was used as a deparaffinization agent [16,29,62]. From our experience, RotiHistol^®^ at room temperature removes paraffin as effectively as xylene; isopropanol also clears sections from paraffin, although only at higher (at 59 °C but not at 39 °C) temperatures. Thus, it seems that it removes only melted paraffin, potentially limiting its application to materials with a melting point below that of paraffin.

Compared with xylene, cedar wood oil, coconut oil and dishwash had inferior staining quality and also cellular architecture (except dishwash) in biological samples [90]. Others reported satisfactory results for cedar wood oil, bleached palm oil [68] and also dishwasher soap (although the temperature should be elevated up to 90 °C, which would not be compatible with some polymers) [75,77], butyldecanoate (temperature up to 60 °C) [69] and PGME [74]. The evaluation of these potential agents is precluded by a lack of uniform evaluation standards. Moreover, plant oils are mixtures of fatty acids, precluding deeper insights into their active components.

Table 1 summarizes before mentioned means to overcome scaffold-related histological challenges.

### 5.6. Staining and Visualization

The FFPE fixation step causes strong protein cross-linking and epitope masking, thus usually requiring antigen retrieval prior to immunohistochemical staining. This procedure employs high temperature and unmasking reagents, such as citrate buffer, to expose epitopes to subsequent antibody binding. Temperature is maintained at about 95 °C [91], thus precluding the application to some polymers (e.g., PCL). However, there are reports that heat-induced antigen retrieval preserved the structure of PCL/PLA and Matriderm scaffolds, likely due to the higher melting temperature of PLA (170 °C), while enzymatic epitope unmasking did not yield immunohistochemical staining and, moreover, pepsin digested the membrane of Matriderm [59]. Enzyme-based digestion is still an alternative solution, which was tested by Fuchs et al. (2019) and demonstrated that treatment with proteinase K provided irregular and insufficient immunostaining, although pepsin treatment worked well on the same cellularized PCL/PLA scaffolds [15]. Still another way is to avoid antigen retrieval at all, for instance, using the HOPE fixation method, which does not utilize formalin and does not cross-link proteins [19].

Longer incubation times and/or concentrations of dyes and antibodies could be required compared with standard cell culture staining protocols due to their potential lower penetration into scaffolds and hydrogels. Moreover, some materials used in scaffold construction may provide nonspecific staining, especially if they contain some biological molecules, such as collagen in gelatin. As gelatin charge depends on acidity (positive at lower pH) [92], negatively charged eosin tends to stain it (our observations). Thus, also useful in numerous applications, gelatin’s ability to infiltrate tissues also poses a risk of affecting staining results. Also, synthetic scaffolds are highly autofluorescent (PCL demonstrates high autofluorescence in the green spectrum); therefore, measures to mitigate it are often applied, such as using confocal microscopy, which also helps visualize deeper into the scaffolds, depending on the architecture and density. Thinner cell-seeded scaffolds could be visualized unsectioned [37,93,94,95]. For example, 50 µm thickness PCL/gelatin scaffolds were imaged with a fluorescent microscope after common immunohistochemical staining [93]. This also applies to the specifically patterned scaffolds (usually containing large 300–700 µm size pores and fibers), as cells, to some extent, could be seen throughout [96,97] or only the top layers are of interest [98].

## 6. Conclusions

Despite the growing use of scaffolds in bioengineering and regenerative medicine, histological processing of these materials still poses challenges across most of the processing steps. These difficulties are primarily related to the scaffold material sensitivity and susceptibility to commonly used chemicals and limited temperature resistance. While standard histological processing protocols used for biological tissues are not always applicable to scaffolds, some modifications are providing effective results for some of the most widely used scaffold materials. Firstly, it is recommended to consider alternative reagents to replace the damaging ones. For example, the existence of numerous xylene substitutes together with the increasing prevalence of automatic tissue processors now enables the avoidance of the use of this toxic chemical in FFPE. Also, one should keep in mind the temperature sensitivity of scaffolds by testing their melting points before processing. Furthermore, the quality of frozen samples may benefit from improved cryopreservation, based on specific properties of the scaffold. Finally, although vibrating microtomy is not widely employed in this field, it holds significant potential for certain scaffold designs. Nevertheless, the lack of standardized protocols for most scaffold materials calls for more comprehensive testing of their compatibility with various histological procedures, as well as for the creation of databases containing validated protocols.

## Figures and Tables

**Table 1:** Summary of studies that applied scaffold-adapted histological processing protocols.

Processing Step	Commonly Used Reagent	Alternative Reagents/Means	Scaffold Material	References
Frozen samples				
Cryoprotection	Gluc, sucrose	5% Gluc, 20% Gluc, 2:1 Gluc:O.C.T., 1:1 Gluc:O.C.T., 1:2 Gluc:O.C.T.	Collagen	[43]
		15% sucrose, 30% sucrose	PEGDMA	[44]
		100% FBS	PEG	[36]
		30% BSA	PEG	[36]
		1% PVA	PEG	[36]
		10% PVA + vacuum	PLGA	[14]
		O.C.T.	PEG	[36]
			PCL/PEO	[37]
		Fisher cryogel	PEG	[36]
		Low-molecular-weight PEGDA	PEGDA	[42]
Formalin-Fixed Paraffin-Embedded samples				
Dehydration	Ethanol	Acetone	PLLA	[57]
Clearing	Xylene	Automatic tissue processor	PCL/PLA	[59]
		HistoClear^®^	PCL	[16]
Impregnation/embedding	Paraffin (melting point ~60 °C)	GMA + vacuum		[13]
		GMA	PCL	[80]
			PLA	[81,82]
			PLLA	[57]
		Technovit^®^ 7100	PCL	[83]
		Low-melting paraffin	PCL/PLA	[15]
		10% gelatin, 25% gelatin	PCL/PLA	[15]
Deparaffinization	Xylene	HistoClear^®^	PCL	[16]

BSA, bovine serum albumin; FBS, fetal bovine serum; Gluc, glucose; GMA, glycol methacrylate; O.C.T., optimal cutting temperature; PCL, polycaprolactone; PEG, polyethylene glycol; PEGDA, polyethylene glycol diacrylate; PEGDMA, poly (ethylene glycol) dimethacrylate; PEO, poly (ethylene)oxide; PLA, polylactic acid; PLLA, poly-L-lactic acid; PVA, polyvinyl alcohol.

## Data Availability

No new data were created or analyzed in this study. Data sharing is not applicable to this article.

## Abbreviations

The following abbreviations are used in this manuscript:

BSA	bovine serum albumin;
DMSO	dimethyl sulfoxide;
FBS	fetal bovine serum;
FFPE	formalin-fixed paraffin-embedded
Gluc	glucose;
GMA	glycol methacrylate;
HA	hyaluronic acid;
HOPE	Hepes-glutamic acid buffer-mediated Organic solvent Protection Effect;
NBF	neutral-buffered formalin
O.C.T.	optimal cutting temperature;
PCL	polycaprolactone;
PEG	polyethylene glycol;
PEGDA	polyethylene glycol diacrylate;
PEGDMA	poly (ethylene glycol) dimethacrylate;
PEO	poly (ethylene)oxide;
PGA	polyglycolic acid;
PLA	polylactic acid;
PLGA	poly (lactide-co-glycolide);
PLLA	poly-L-lactic acid;
PPy	polypyrrole
PVA	polyvinyl alcohol;
SF	silk fibroin.
